# Colloidal Lignin Particles and Epoxies for Bio-Based, Durable, and Multiresistant Nanostructured Coatings

**DOI:** 10.1021/acsami.1c06087

**Published:** 2021-07-15

**Authors:** Karl Alexander Henn, Nina Forsman, Tao Zou, Monika Österberg

**Affiliations:** School of Chemical Engineering, Department of Bioproducts and Biosystems, Aalto University, Vuorimiehentie 1, 02150 Espoo, Finland

**Keywords:** lignin, lignin nanoparticles, nanostructured, epoxy, bio-based, surface coating

## Abstract

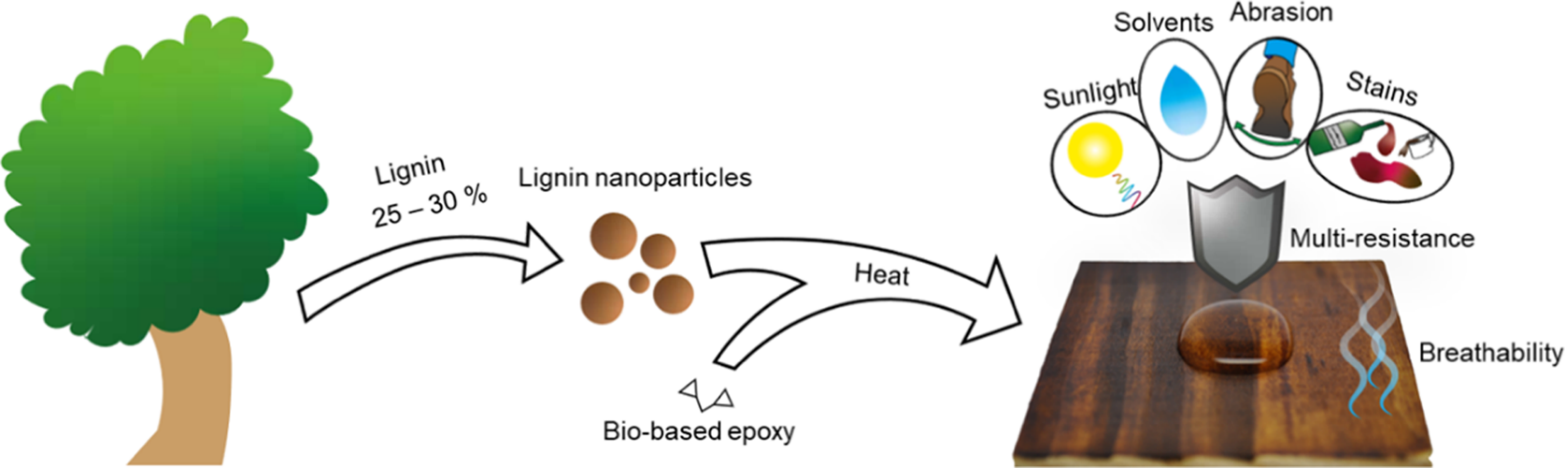

There is a need for safe and sustainable alternatives in the coating industry. Bio-based coatings are interesting in this perspective. Although various oils and waxes have been used as traditional wood coatings, they often lack sufficient durability. Lignin is an abundant natural polyphenol that can be used to cure epoxies, but its poor water solubility has impeded the use of unmodified lignin in coatings in the past. To address this issue, water-dispersible colloidal lignin particles (CLPs) and an epoxy compound, glycerol diglycidyl ether (GDE), were used to prepare multiprotective bio-based surface coatings. With the GDE/CLP ratios of 0.65 and 0.52 g/g, the cured CLP–GDE films became highly resistant to abrasion and heat. When applied as a coating on wooden substrates, the particulate morphology enabled effective protection against water, stains, and sunlight with very thin layers (less than half the weight of commercial coatings) while retaining the wood’s breathability excellently. Optimal hydrophobicity was reached with a coat weight of 6.9 g(CLP)/m^2^, resulting in water contact angle values of up to 120°. Due to their spherical shape and chemical structure, the CLPs acted as both a hardener and a particulate component in the coating, which removed the need for an underlying binding polymer matrix. Light interferometry measurements showed that while commercial polymeric film-forming coatings smoothened the substrate noticeably, the particulate morphology retained the substrate’s roughness in lightweight coatings, allowing for a high water contact angle. This work presents new strategies for lignin applications in durable particulate coatings and their advantages compared to both currently used synthetic and bio-based coatings.

## 1 Introduction

Coatings and paints are essential for protecting and repairing surfaces^1^ and to enhance sustainability by prolonging the lifetime of products. The economic and ecological significance of good anticorrosion coatings is immense. In China, for example, the cost of corrosion was 310 billion USD in 2015.^2^ While anticorrosion measures will always remain economically valuable, the significance of wood coatings will likely see an increase. Due to the global efforts to meet sustainability standards, many countries are currently replacing or planning to replace concrete with wood in buildings.^3^ However, because wood is prone to degradation when exposed to sunlight and moisture,^4,5^ protective coatings will be increasingly needed to enable greater use of wood in buildings. These coatings should preferably be sustainable, and plant-based solutions could be a step in this direction.

Current protective coatings for materials such as wood, concrete, metals, and composites are petroleum-based, including, *e.g.*, polyurethanes, epoxies, polyphenols, acrylic resins, and polyamides. Their mechanical, chemical, and thermal durability as well as their versatility and reasonable price make these coatings attractive.^6^ Vegetable-oil coatings such as tall linseed, coconut, soybean, and castor-oil coatings can be more sustainable alternatives.^6,7^ These oils are often combined with synthetic resins and cross-linkers to improve their properties, and there are very few coating applications without chemical modification.^6^ Legislative action is pushing the coating industry towards safety and sustainability, which creates a demand for alternatives to the currently available options. For example, the amount of volatile organic compounds (VOCs) has been regulated due to their detrimental impact on not only health but also the ozone layer.^1^ The European Union (EU) has restricted some chemicals often used by the coating industry such as bisphenol A and formaldehyde (used in epoxy and polyurethane coatings). The EU also recently classified titanium dioxide, one of the most widely used pigments in paints, as a class II carcinogen.^8^ These types of restrictions and reclassifications will inevitably lead to changes in the current coating formulations.

Achieving low cost, safety, and high performance in bio-based coatings is crucial to be able to apply them commercially, but finding raw materials that can fulfill these criteria is challenging. One potential material is lignin, which is an amphiphilic natural polymer present in wood and other plant sources. Despite its abundance, it is often regarded as a waste product of pulping and biorefinery processes. Each year, about 60–120 million tonnes of lignin is isolated worldwide, 98% of which is incinerated for energy recovery.^9^ Lignin possesses several useful properties, such as antioxidant activity, UV absorption, and relatively high resistance against microbial degradation.^10−15^ However, the poor solubility of most lignin types and the mediocre performance of lignin-based products have so far limited its commercial applications. Nonetheless, some successful coatings of chemically modified lignin have been developed.^16−21^ Carlos de Haro et al.^17^ presented a corrosion-resistant coating of silanized, THF-fractionated, lignin for aluminum substrates, and Hajirahimkhan et al.^18^ presented UV-curable, methacrylated kraft lignin coatings with good substrate adhesion properties and thermal stability. Park et al.^22^ developed phenol-formaldehyde coatings with 10–40% acetylated lignin, which possessed good water-barrier properties. However, the chemical modification needed makes these approaches less sustainable and more expensive.

In a recent study by Li et al.,^23^ unmodified kraft lignin was successfully used together with the epoxy compound, glycerol diglycidyl ether (GDE), as a water-based wood adhesive. However, the authors reported that spreading the mixture of GDE, lignin, and water was difficult before milling the lignin into smaller particles. In this respect, the use of water-dispersible spherical colloidal lignin particles (CLPs) could be advantageous since the dispersion spreads easily.^24^ CLPs, also called lignin nanoparticles, can be prepared in various sizes and are highly customizable.^24−26^ Although kraft lignin itself is poorly water-soluble, CLPs can be used in water-based systems, such as biopolymer blends and emulsions.^11,27^ Sipponen et al.^28^ showed that CLPs can also be used as vectors for hydrophobic substances in water-based systems, which could enable the use of non-water-soluble cross-linkers in a water dispersion of CLPs. Nevertheless, the application of CLPs as a nanostructured coating is yet unexplored.

Micro- and nanostructured coatings have gained attention because of their often excellent anticorrosion, antibacterial, anti-icing, and UV-shielding properties.^29^ The high surface roughness of nanostructured coatings is one important factor contributing to their exceptional hydrophobicity.^30,31^ The sol–gel and layer-by-layer methods are examples of useful and relatively scalable methods to create hierarchical nanostructures.^32−35^ Particulate coatings are a type of structured coating where the structures are established by micro- or nanoparticles. One example of bio-based particulate coatings is the layer-by-layer approach to attach wax particles onto wood, textiles, or other cellulosic substrates using a cationic polymer or particle as binder.^30,36−38^ Electrostatically bound coatings such as these can be used extremely sparingly while providing excellent hydrophobicity. They are easy to apply but can be washed away by detergents.^36^ The resistance against washing and abrasion can be increased by covalently binding particles to a polymer matrix or by embedding the particles in a polymer matrix bound to the substrate. This makes the coating thicker and possibly more expensive but improves the coating’s adhesion to the substrate.^29,39,40^ Nanostructured coatings are often developed for very specific applications where hydrophobicity and water repellency are the main targets^29,41^ although their commercial use is still limited. Furthermore, studies on nanostructured coatings seldom include information about mechanical or chemical durability, both of which are important properties in practice. To date, most nanostructured coatings reported are based on different types of metal oxides, e.g., zinc, titanium, or silicon oxide,^42−44^ but their safety has recently become a concern. Due to the harmful effects of the inhalation of zinc oxide nanoparticles,^45,46^ the EU has implemented restrictions for products where particles can reach the lungs of users.^47^ Likewise, the use of silicon dioxide particles is restricted as they are known to cause silicosis and have been linked to cancer.^8^ Titanium dioxide is also restricted due to its carcinogenicity. While metal oxide particles are prone to generate reactive oxidative species (ROS) in living cells, it is noteworthy that CLPs possess antioxidant properties.^10,11^ This is a major advantage, as the generation of ROS is believed to be one of the most significant causes of the metal oxide particles’ cytotoxicity.^48^ Due to their spherical structure, water dispersibility, and inherent UV resistance and radical scavenging properties, CLP could have significant potential as functional particulate components in coatings.

Inspired by the advantages of particulate coatings, on the one hand and CLPs, on the other hand, this paper presents a method to utilize the amphiphilic properties of CLPs for the preparation of safe, water-based, and durable particulate surface coatings without the use of a binding polymer matrix. Because GDE is poorly soluble in water, it cannot be used in regular water-based coatings without nonpolar solvents. Nevertheless, by combining CLPs and GDE, an aqueous surface coating using lignin, free of volatile organic solvents, can be prepared. The coating demonstrates good resistance to abrasion, solvent, water, and UV light while having outstanding breathability compared to the commercial oil, lacquer, and epoxy coatings that were examined and can be applied onto a wide range of rigid materials, as demonstrated by its application on wood and metal surfaces.

## 2 Experimental Section

### 2.1 Materials

The lignin used in this study was Biopiva 100 kraft lignin (UPM, Finland). Etax A (≥94.0%) ethanol was purchased from Altia Industrial. AnalaR NORMAPUR (≥99.5%), tetrahydrofuran (THF), and acetone were purchased from VWR Chemicals BDH. Technical-grade glycerol diglycidyl ether, dimethylformamide (99.8%), pyridine (99.8%), *N*-hydroxy-5-norbornene-2,3-dicarboxylic acid imine (97.0%), chromium(III) acetylacetonate (≥98.0%), 2-chloro-4,4,5,5-tetramethyl-1,3,2-dioxaphospholane (95%), and chloroform-D (99.8%) were purchased from Sigma-Aldrich. All chemicals were used as received.

### 2.2 Preparation of Lignin Nanoparticles

A solution of 30.7 wt % of ethanol, 34.6 wt % of analytical-grade THF, 30.0 wt % of deionized water, and 4.7 wt % of BioPiva 100 kraft lignin was stirred for 3 h in a closed flask and vacuum-filtered using 589/3-grade ashless filter papers (Schleider & Schuell). The solution was then swiftly added to 1.72 times its mass of rapidly stirred deionized water, which initiated the precipitation of water-dispersible nanoparticles. The dispersion was stirred for 15 min and then rotary-evaporated at 40 °C and 30 mbar until the THF and ethanol were removed. The solvent-free CLP dispersion could be concentrated by centrifugation, where 45–50 mL of dispersion was centrifuged at 10 500 rpm for 30 min in Eppendorf tubes. The supernatant was removed, and the particles were redispersed by vortexing.

### 2.3 Particle Size Characterization

The particle size was determined by dynamic light scattering using a Zetasizer Nano-ZS90 (Malvern, U.K.) instrument. The surface charge was measured using a zeta dip cell, and the ζ-potential values were calculated from the obtained electrophoretic mobility data using the Smoluchowski model. Dynamic light scattering particle size distribution data can be found in Figure S2.

### 2.4 ^31^P-NMR Characterization

Two measurements were performed separately. The lignin sample was dried in a vacuum oven at 30 °C overnight. Then, 30 mg of the dried lignin was weighed into a glass vial. One hundred and fifty microliters of dimethylformamide, 100 μL of pyridine, 200 μL of *N*-hydroxy-5-norbornene-2,3-dicarboxylic acid imine (10.2 μmol), functioning as the internal standard, and 50 μL of chromium(III) acetylacetonate were added in that order. One hundred and fifty microliters of 2-chloro-4,4,5,5-tetramethyl-1,3,2-dioxaphospholane, as the phosphorylating agent, was then added dropwise with approximately 2 s between each drop while stirring with a magnetic stirrer. Finally, 300 μL of chloroform-d was added, and the stirring continued for ca. 10 min.

The ^31^P-NMR spectra were analyzed using a Bruker Avance 400 MHz spectrometer (MA). One hundred and twenty-eight scans were performed using the pulse sequence *zgig* with a pulse angle of 90°, an acquisition time of 1 s, and a pulse delay of 5 s. The results are found in Table S2.

### 2.5 Surface Coating Sample Preparation

Glycerol diglycidyl ether (GDE) was used as an epoxy compound without modification. The molar amount of hydroxyl groups per mass of kraft lignin (using an average of values obtained by us and by previous studies on the same lignin^49^) and the molar amount of epoxide groups per mass of GDE were used to calculate the GDE/CLP mass ratio, where the molar ratio of epoxy groups to hydroxyl groups is approximately 1:1. The GDE/CLP ratios that were evaluated in this study were between 1.4 and 0.6 times the calculated equimolar ratio, which resulted in GDE/CLP mass ratios of 0.90, 0.78, 0.65, 0.52, and 0.39 g/g.

To prepare the coatings, aqueous 20 wt % of CLP dispersions were prepared and mixed with GDE according to the desired GDE/CLP ratios. The mixtures were stirred using a magnetic stirrer for 1–2 min, whereafter they were coated onto the substrates. Pinewood samples of dimensions 6.2 cm × 6.3 cm × 2 cm were used as substrates for all tests except scanning electron microscopy for which birch ply sheets were used. The wooden substrates were premoistened with a small amount of deionized water to improve spreading efficiency and avoid the formation of uneven spots. The CLP concentration was kept between 10 and 20 wt % before adding the desired amount of GDE to avoid rapid curing (and hence the formation of aggregates) as well as the separation between the GDE and the aqueous phase.

Stainless steel metal disks (Taber Industries, Steel, S-16) measuring 10 cm x 10 cm were used as metal substrates. When coating metal samples, 1 g of 20 wt % CLP dispersions was mixed with GDE in the previously mentioned ratios for 1−2 minutes. The mixture was spread onto the plates in portions of 200 μL at a time. The metal plates were kept at 70 °C while the coating was spread. The coated substrates were then cured at 105 °C. All coatings on wooden substrates were cured for 90 min, while the coatings on metal substrates were cured for 20–100 min.

For thermal analysis and curing monitoring, 500–1000 μL of 20 wt % CLP dispersions was mixed with GDE according to the desired GDE/CLP ratios. The mixture was then added to an aluminum pan and cured at 105 °C. The curing time was 60 min for thermal analysis and 0–100 min for Fourier-transform infrared (FTIR) absorption curing monitoring.

The commercial and water-based coatings Tikkurila KIVA 70 wood lacquer (Tikkurila, Finland), Teknos Woodex bioleum wood oil (Teknos, Finland), and Solmaster EP10 epoxy coating (Solmaster, Finland) were used as references for all tests and were applied to the substrates according to the manufacturer’s instructions, except that only one layer of the referential epoxy coating was applied to wooden substrates for breathability tests instead of the two layers recommended by the manufacturer. All chemicals in the work were used as received.

### 2.6 Thermal Characterization

Samples of GDE/CLP ratios of 0.65, 0.52, and 0.39 g/g were prepared as previously described. The kraft lignin reference was heated for 60 min at 105 °C before analysis for increased comparability between it and the cured CLP–GDE samples. The thermal properties of the samples were analyzed using a DSC 6000 (PerkinElmer, MA) differential scanning calorimeter. Pierced aluminum sample crucibles were used for the analysis. The program was set to heat from 50 to 250 °C at a rate of 5 °C per minute. Before the start of each thermal scan, an isothermal step that lasted 3 min occurred. Thermogravimetric analysis was conducted using a Q500 thermogravimetric analyzer (TA instruments, Delaware) using a heating rate of 10 °C per minute in nitrogen from 30 to 700 °C. Thermal heat resistance indexes (*T*_HRI_) were calculated according to eq 1
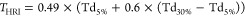
1where Td_5%_ and Td_30%_ are the temperatures at which 5 and 30% of the initial mass of the sample have been lost, respectively.

The main volatile gases that were released by the thermal degradation were analyzed using an STA 449 F3 Jupiter device coupled to a QMS 403 Aëolos Quadro (Netzsch, Germany). The measurement was performed with a heating rate of 5 °C per minute in helium from 40 to 550 °C. The molecules with atomic mass units of 15, 16, 18, 28, and 44 were occurring most prevalently and were determined to correspond to CH_3_, CH_4_, H_2_O, CO, and CO_2_, respectively.^50,51^

### 2.7 Microscopy

The coatings, prepared as described in section 2.5 on metal plates and cured for 60 min, were analyzed using a Multimode 8 AFM with a Nanoscope V controller (Bruker, Santa Barbara, CA) using NCHV-A probes (antimony-doped silicon with an 8 nm tip radius) in tapping mode in air. Roughness values for each sample were obtained from one image of 10 μm × 10 μm.

The coatings were also analyzed using a Phenom Pure G5 scanning electron microscopy (SEM) with a standard sample holder (Thermo Scientific, MA). Before analysis, the samples were coated with a gold–palladium mixture (Au80Pd20) with a Q 150R S plus rotary-pumped coated (Quorum Technologies, U.K.) using a sputter current and time of 20 mA and 20 s, respectively, and a tooling factor of 1.00.

### 2.8 Breathability and Hydrophobicity

The breathability of the coatings was evaluated using the NORDTEST method.^52^ Briefly, all sides of the wooden samples (with dimensions 2 cm × 6.2 cm × 6.3 cm) except the coated surfaces were covered with aluminum tape and placed in a Rumed 4201 (Rubarth Apparate GmbH, Germany) climatic chamber set to cycle between 33% RH (16 h) and 75% RH (8 h) at 23 °C. The samples were conditioned for 48 h in the test setting before commencing the test. Then, the samples were weighed 5–10 min before each change in humidity for 3 days. The moisture buffering value was obtained from the average mass change per area per change in relative humidity.

The hydrophobicity and water absorbance were evaluated by water contact angle and volume change measurements of MilliQ water droplets on surfaces using a ThetaFlex Tensiometer (Biolin Scientific, Sweden). The droplet size was 4 μL, and the contact angle was measured 1 min after the drop had been placed onto the substrate when the droplet had stabilized on the substrate.

### 2.9 Sunlight Resistance

The coatings’ sunlight resistance was evaluated on wooden samples. A Suntest CPS+ device (Atlas Material Testing Technologies, IL) with a xenon lamp shining at 765 W/m^2^ was used to simulate sunlight. According to data from the European Union’s Photovoltaic Geographical Information System^53^ (PVGIS-SARAH database), the daily average horizontal irradiance in the Helsinki region in Southern Finland is between 104 and 117 W/m^2^, resulting in a total energy input of 2.5–2.8 kWh (24 h total irradiance). According to this data, the device provides the same energy input in the form of light about 6.4–7.4 times faster than in real conditions. The samples were exposed to 25 days of simulated sunlight (which equals 5.3–6.1 months in real conditions). The samples were photographed in a photochamber with a standard lighting setting before the test and after 1, 3, 5, 7, 10, 15, 20, and 25 days of exposure. The color changes where then examined computationally. The images were blurred to even the colors, thus decreasing errors caused by differences in individual pixels. The color change was calculated from the average change in RGB color coordinates from 3 to 5 different spots. The cumulative isolated and combined color change were calculated according to eqs 2 and 3, respectively
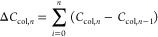
2
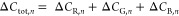
3where *C*_(col)_ represents the color coordinate for red (R), blue (B), or green (G), *n* represents the number of the measurement, and Δ*C*_tot,*n*_ represents the total color change in the *n*th measurement.

### 2.10 Abrasion Resistance

The abrasion resistance of the coatings was evaluated using a Taber Abrader (Taber Industries, NY), following the ASTM-D4060 method.^54^ Briefly, the coated metal plates were conditioned at 23 °C and a 50% relative humidity for 24 h before the evaluation. Next, the samples were weighed and abraded with a Taber Abrader (Taber Industries) with a load of 1000 g and CS-10 abrasive wheels. The abrasion was stopped before tearing through the coating, whereafter the samples were dusted off and weighed again. The abrasion resistance index was expressed as the change of mass per cycle.

### 2.11 Fourier-Transform Infrared Analysis

A spectrum two FTIR Fourier-transform infrared (FTIR) spectrometer (PerkinElmer, MA) was used to analyze the epoxidized lignin and the curing kinetics of the GDE-CLP mixtures. The resolution was set to 1 cm^–1^, and 40 scans were performed for each measurement. All measurements were performed on dried samples. Attenuated total reflection (ATR) was corrected for the measured data, and the spectra were normalized using Spectrum 10 software (PerkinElmer) between wavenumbers 3500 and 500 cm^–1^. The consumption of epoxide groups by the curing reaction was analyzed by comparing the integrated intensities of the band between 880 and 930 cm^–1^ at 0, 5, 10, 20, 30, 40, 50, 60, 70, 80, 90, and 100 min of curing at 105 °C. Mixtures of noncured GDE and CLPs were air-dried before the measurement to avoid initiating curing, while the other samples were first dried while curing at 105 °C and then air-dried if moisture remained.

### 2.12 White Light Interferometry to Determine Surface Roughness

The roughness of wooden substrates coated with GDE/CLP and reference coatings was analyzed using a ContourGT-K scanning white light interference microscope (Bruker, MA). The samples were scanned in VXI-mode, and the arithmetical mean height (*S*_a_) and the root-mean-square height (*S*_q_) were calculated using Vision64 Map software, and averages were calculated from three measurements. The area scanned was 315 μm × 236 μm, the focus was 20 × 1x, and the pixel size was 0.493 μm.

### 2.13 Solvent and Stain Resistance

Wood substrates coated with GDE/CLP coatings and reference coatings were stained with 45 μL of wine and coffee and 2 × 45 μL of analytical-grade acetone (with 20–30 s between the two additions). Acetone was chosen for the solvent resistance test as it dissolves the kraft lignin used herein and due to its vast use in domestic and industrial applications. The liquids were allowed to remain on the substrates for 1 min, after which they were removed by pressing with a paper towel. Photos were taken before, during, and after the staining in a photochamber with a standard lighting setup.

## 3 Results and Discussion

### 3.1 Preparation of Cross-Linked Lignin Nanoparticle Coating

The coatings were prepared using aqueous dispersions of CLPs mixed with GDE in various ratios and concentrations. The CLPs decreased the separation between GDE and the aqueous phase in a concentration-dependent manner. Sufficiently high concentrations were needed to avoid phase separation, but too high CLP concentrations lead to very rapid curing and aggregation. To address both issues, dispersions with concentrations between 10 and 20 wt % were found to be optimal.

**Figure 1:**
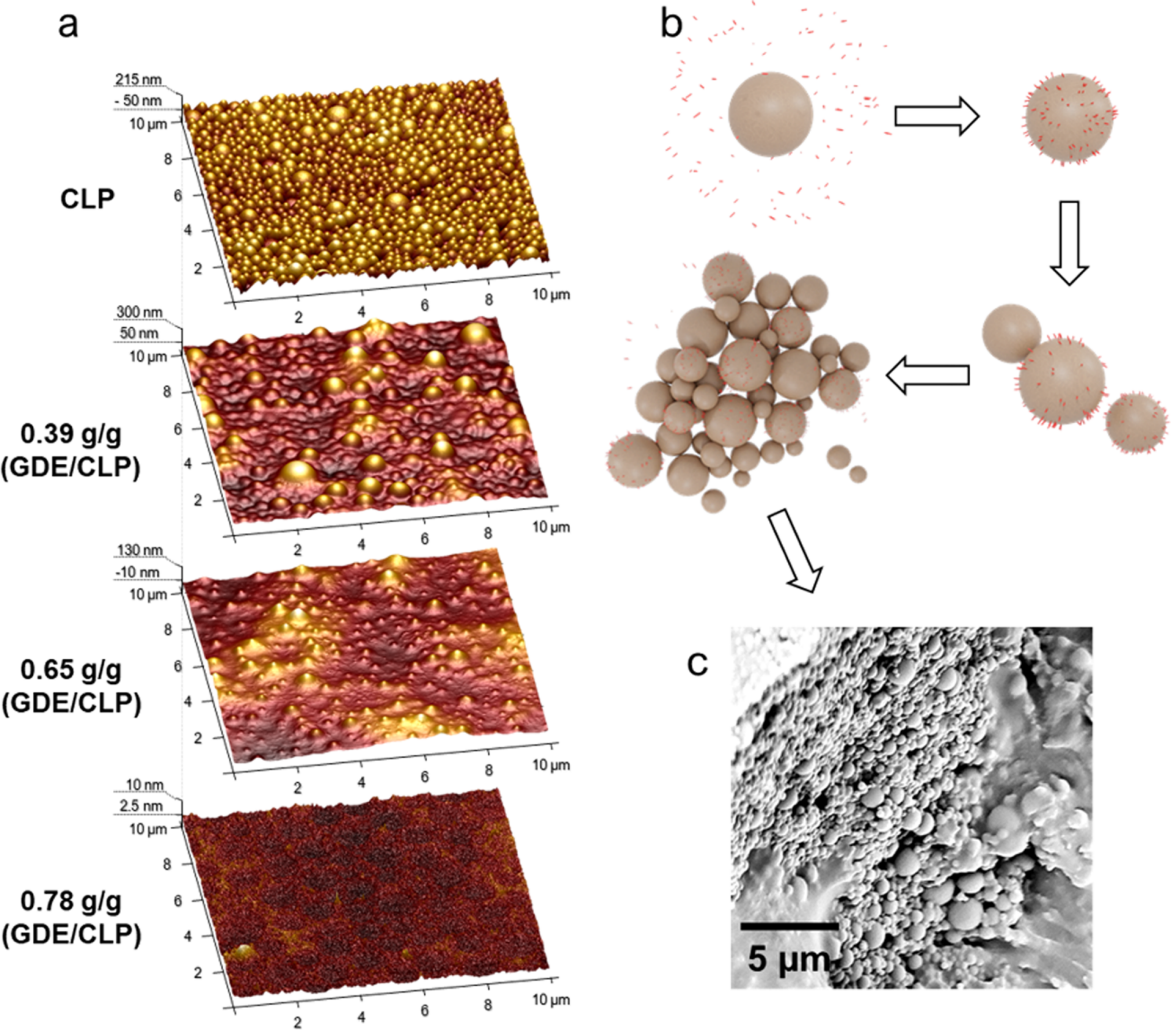
Formation and appearance of CLP networks cross-linked by GDE. (a) AFM images of a layer of only CLPs and coatings with increasing GDE/CLP ratios from 0.39 to 0.78 g/g on metal substrates. (b) Proposed coating formation mechanism. The formation of CLP networks begins when GDE is adsorbed onto the CLPs, which enable them to attach to each other and create networks of linked particles as more particles are attached. (c) SEM image of a coating with a GDE/CLP ratio of 0.65 g/g. All samples were cured before the measurements.

**Figure 2:**
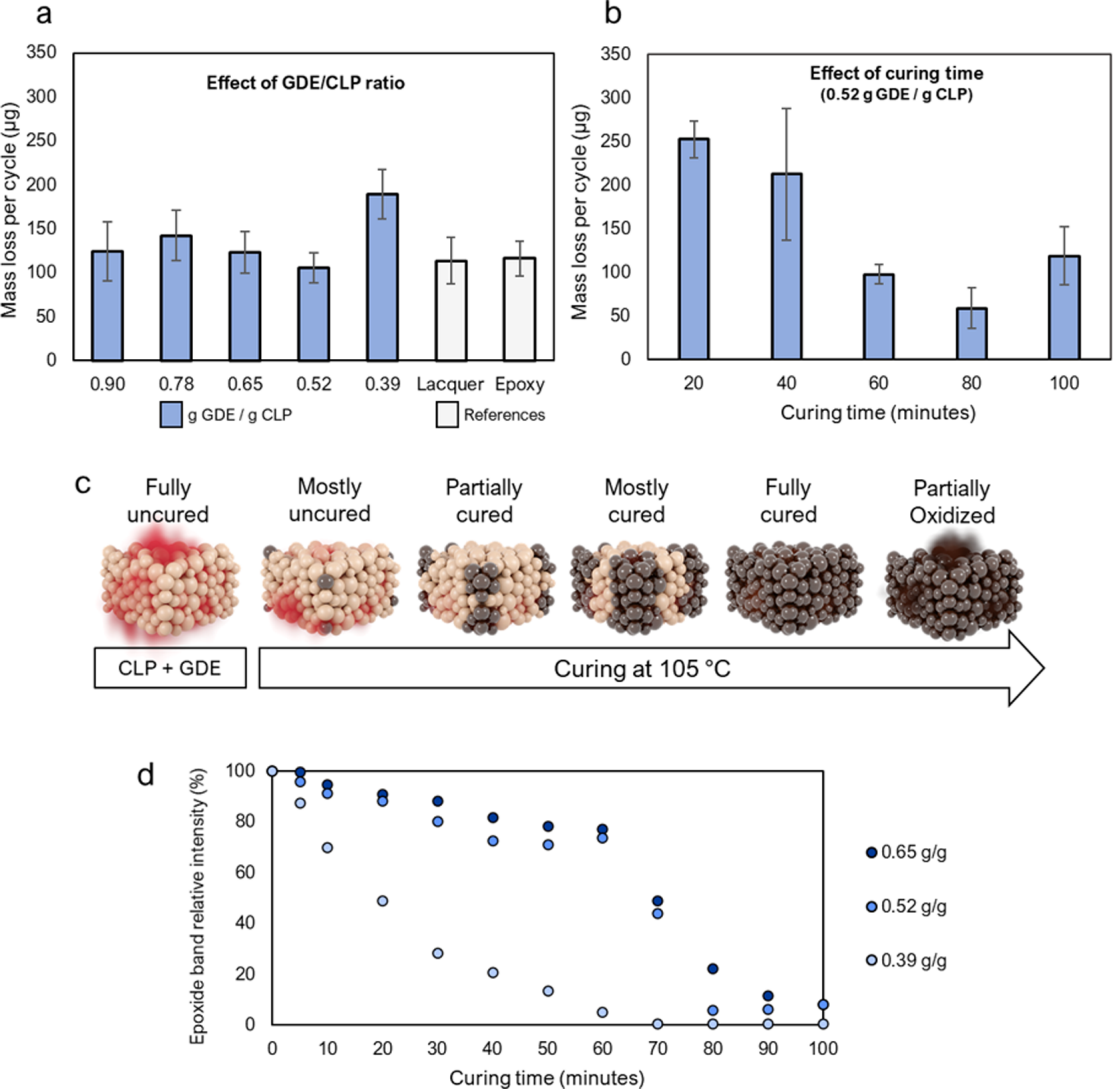
Effect of the GDE/CLP ratio on the abrasion resistance and curing kinetics of the coatings. (a) Abrasion resistance of GDE/CLP coatings with different GDE/CLP ratios and reference coatings. (b) Abrasion resistance of coatings with a GDE/CLP ratio of 0.52 g/g cured for different lengths of time. (c) Illustration of the curing procedure. (d) Intensity change of the FTIR epoxide absorption signal between wavenumbers 930 and 880 cm^–1^.

The CLP concentration’s effect on the reduction in phase separation between the dispersion and GDE strongly suggests that GDE is adsorbed onto the CLPs. This is seen in the AFM images of coatings with varying GDE/CLP ratios (Figures 1a and S1a). The excessive GDE in the coatings with a GDE/CLP ratio of 0.78 g/g could have either dissolved the particles or, alternatively, covered the particles to such a degree that they could not be distinguished. Particles in coatings with a GDE/CLP ratio of 0.65 g/g were, on the other hand, clearly distinguishable, despite the slight decrease in the particles’ shape definition. In accordance, the roughness values obtained from the GDE/CLP coatings in Figure 1 decreased with increasing GDE/CLP ratio (Table 1). Scanning electron microscope (SEM) images of the same sample (Figure S1b) show that most of the particles remained undissolved, indicating that the decreased roughness is most likely due to excess GDE, which surrounds and coats the particles. Assuming that GDE is mostly adsorbing onto the particles rather than absorbing into them, the particle size would affect the optimal GDE/CLP ratio. The average particle hydrodynamic diameter in this study was determined to be 450 nm using dynamic light scattering measurements (Figure 2S).

**Table 1:** Effect of GDE/CLP Ratio on Surface Roughness Measured by AFM and Reported as the Average Height (*R*_a_) and Root-Mean-Square Height (*R*_q_) Measured on Metal Plates Coated with Multiple Layers of Coating^a^

GDE/CLP ratio (g/g)	*R*_a_ (nm)	*R*_q_ (nm)
0 (CLP)	40.2	50.5
0.39	46.4	62.2
0.65	19.3	24.7
0.78	0.8	1.1

a (*n* = 1 × 10 μm^2^).

Based on these observations, a mechanism for the network formation was proposed, as illustrated in Figure 1b. When the coating is dried and the water content is decreased, capillary forces drive the particles closer to each other, which enables linking and consequently the formation of particle networks.

Usually, particulate coatings contain a polymer matrix phase which acts as a binder for the particulate phase that is preferentially dispersed mostly on top of the matrix.^39^ Electrostatic binders, which are useful for layer-by-layer coatings, can alternatively be used in which case the particles can be, *e.g.*, sprayed or dip-coated onto the desired surface. In these cases, the particulate phase is not covalently bound to the surface and can be detached by abrasion.^30,55^ When polymer binders are used, it is often epoxy, although many other types of polymer matrices are also used. This strategy is accompanied by the challenge of controlling the distribution of the particulate phase as the coating cures/dries since its arrangement will affect the coating’s mechanical and surface properties.^39^ In our case, the CLPs act as both a hardener and a particulate component. This results in high mechanical strength and diminished need to control the distribution of particles, and this strategy is highly beneficial for the preparation of the coating as it is done just like any commercial epoxy coating. The easy preparation allows the coating to be prepared by laymen in their homes as well as using current industrial processes.

### 3.2 Abrasion Resistance and Curing Efficiency

Since many coatings are exposed to eroding or abrading factors, resistance against mechanical abrasion is important for protective coatings. Therefore, the abrasion resistance as a function of the GDE/CLP ratio and curing time was evaluated on metal substrates (Figure 2). A GDE/CLP ratio of 0.52 g/g possessed the highest abrasion resistance of the compared ratios, with an average mass loss/cycle of 106 μg. This value is similar to those of the commercial epoxy coating (Solmaster EP10) and lacquer (Tikkurila KIVA 70), which both reached mass loss values of approximately 115 μg/per cycle. The coatings with higher GDE/CLP ratios (0.65–0.90 g/g) reached values between 125 and 140 μg/cycle. The small decrease in abrasion resistance in samples with an excessive GDE/CLP ratio suggests that unreacted GDE may act as a softener. A GDE/CLP ratio of 0.39 g/g, which in contrast contained an excess of lignin, possessed a weak abrasion resistance of 190 μg/cycle, likely due to incomplete CLP-network formation.

Since a GDE/CLP ratio of 0.52 g/g performed best in the abrasion tests, the effect of the curing time at 105 °C was evaluated using this ratio (Figure 2b). Increasing the curing time from 60 to 80 min resulted in an increase of 40% in the abrasion resistance. However, increasing the curing time further to 100 min decreased the abrasion resistance. To find the reason for this behavior and optimize the coating, the kinetics of the curing reaction was investigated by monitoring the decrease in the Fourier-transform infrared absorption (FTIR) signal at 930–880 cm^–1^, which is characteristic of oxirane (epoxide) groups (Figure 2d). The results show that the band at 911 cm^–1^, caused by the C–O stretching of the oxirane groups in GDE, disappears after 70 min of curing for a GDE/CLP ratio of 0.39 g/g. In the GDE/CLP ratios of 0.52 and 0.65 g/g, the absorption band at 911 cm^–1^ does not completely disappear but reaches minimum values of 6% and 8% of their original values after 80 and 100 min of curing, respectively. These differences were expected. Although the cross-linking reaction should, in theory, proceed to completion when the epoxide:hydroxyl ratio is 1:1, the increase in rigidity caused by the curing constrains the completion of the reaction due to the restricted movement of molecules. Additional data of the curing monitoring by FTIR and the full absorbance spectra of a GDE/CLP coating with a ratio of 0.52 g/g cured for different lengths of time are found in the Supporting Information (Figures S3 and S4).

The decrease in abrasion resistance observed upon increasing the curing time from 80 to 100 min could be due to oxidative reactions, ultimately making the coating brittle (Figure 2c). This is supported by the FTIR spectra (Figure S4), which showed that samples heated for over 20 min often showed an intense band at 2350 cm^–1^, corresponding to carbon dioxide, when measured immediately after being removed from the heating.

Both epoxide groups of the GDE molecules must react for the oxirane band at 911 cm^–1^ to disappear. The GDE/CLP ratios of 0.52 and 0.65 g/g may contain some amount of once-reacted GDE molecules that are incapable of reaching reactive groups and therefore remain visible in the FTIR spectra.

The results show that the durability is significantly affected by the degree of curing, which is affected by the GDE/CLP ratio and the curing time. The durability is optimized when the amount of GDE is high enough to fully cross-link the CLPs but does not leave excess GDE in the network. The curing time should be sufficient for the reaction to proceed to completion, but unnecessarily long curing times can lead to heat-induced oxidative reactions causing brittleness.

### 3.3 Water Repellency of Coatings

**Figure 3:**
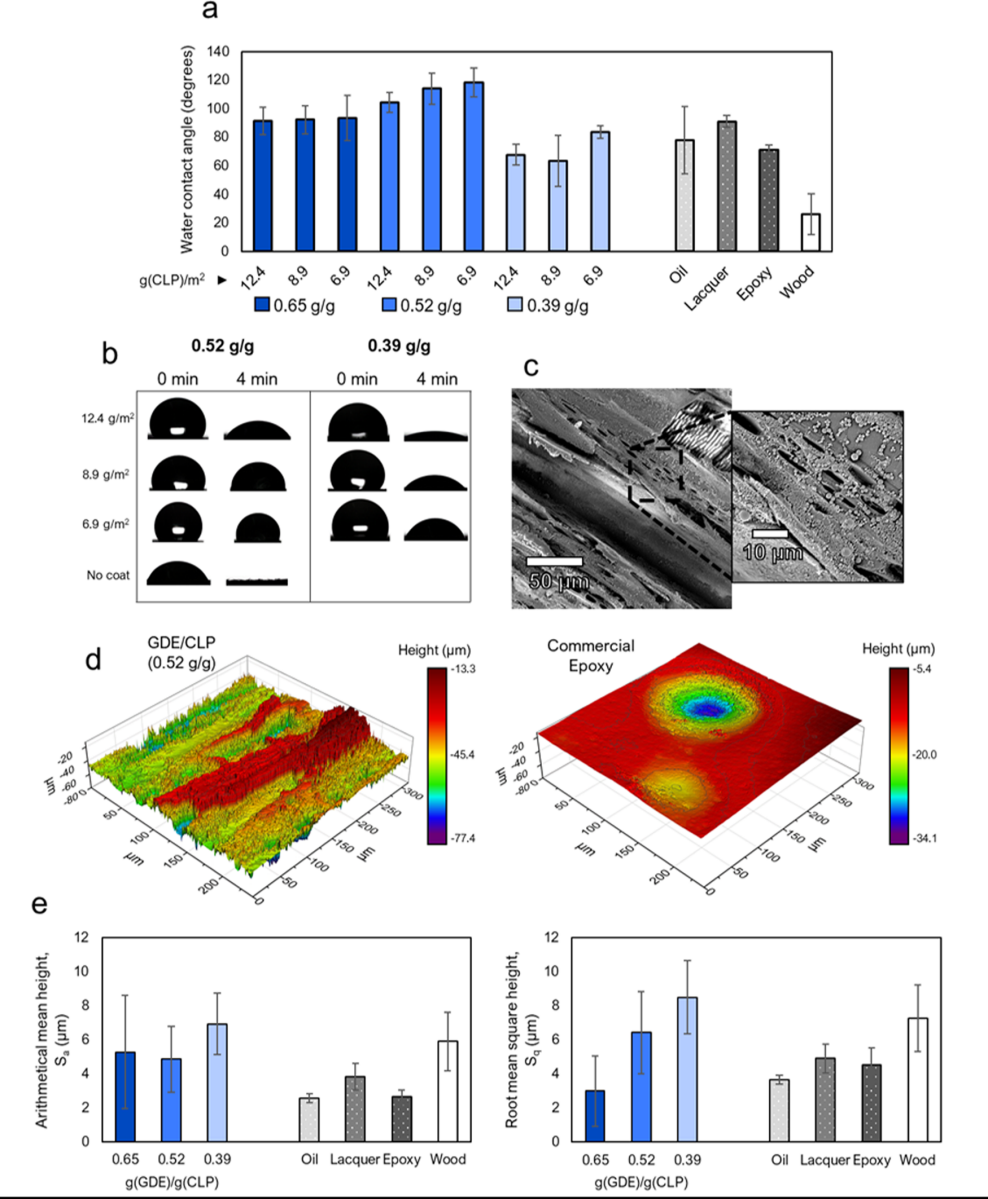
Effect of GDE/CLP ratio, coat weight, and surface roughness on water resistance of the wood coatings. (a) WCAs of GDE/CLP coatings of different thicknesses, commercial reference coatings, and uncoated wood after 60 s of surface contact. (b) Change of droplet size on GDE/CLP coatings and uncoated wood over time. (c) SEM image of a coating with the respective GDE/CLP ratio and thickness of 0.52 g/g and 12.4 g(CLP)/m^2^. (d) Light interferometry height color-map images of a coating with a GDE/CLP ratio of 0.52 g/g (left) and a commercial epoxy coating (right). (e) Surface roughness values obtained by light interferometry measurements of wood coated with different GDE/CLP ratios with coat weights of 12.4 g(CLP)/m^2^, commercial nonparticulate coatings, and uncoated wood.

Although many industrial coatings are used primarily to protect against physical deterioration and wear, anticorrosive metal coatings and wood coatings are required to repel water as well. Due to its many polar structures,^19,56,57^ lignin is rather hydrophilic in nature. Water contact angles (WCAs) of lignin-containing coatings are commonly around 80°,^17,19,56−58^ while 90° is commonly considered the limit to classify a surface as hydrophobic. Herein, we quickly observed that while coating layers of more than 20 g(CLP)/m^2^ fully blocked water from absorbing into wooden substrates, thinner layers created larger WCAs. To find the minimum efficient coat weight (mass per area), WCA and water absorption measurements (Figure S5) were performed on wood with three GDE/CLP ratios of coat weights in the range of 12.4–6.9 g(CLP)/m^2^ (Figure 3).

The GDE/CLP ratio was more significant than the coat weight for the WCA. A GDE/CLP ratio of 0.52 g/g produced WCAs between 105 and 118° and was hence the most hydrophobic of the three evaluated ratios. These values are exceptional for lignin-based coatings,^17,19,56−58^ especially when considering the high lignin content (66% for a GDE/CLP ratio of 0.52 g/g). A GDE/CLP ratio of 0.65 g/g achieved WCAs between 91 and 93°, which is still high compared to the references and just above the limit of 90° to be considered hydrophobic. The coat weight had a less clear effect on the hydrophobicity, but for the 0.52 g/g GDE/CLP sample, a slight increase in WCA was observed with decreasing coat weights. These results can be explained by the combination of surface chemistry and roughness. As already seen with respect to abrasion resistance (Section 3.2), a GDE/CLP ratio of 0.39 g/g is too low to efficiently cross-link the CLPs, and the hydrophilicity of the lignin thus decreases the WCA in this case. For the GDE/CLP ratios of 0.52 and 0.65 g/g, the low surface energy of GDE is dominating, and hydrophobic coatings were formed even at the lowest tested coat weight.

The surface roughness was examined by white light interferometry (Figure 3e) to correlate WCA and surface roughness. The GDE/CLP coatings had a large margin of error, similar to that of wood, likely because the heterogeneity of the wood substrate affected the results for the thin coatings. High GDE/CLP ratios decreased the surface roughness. The obtained arithmetical mean heights (*S*_a_) of all GDE/CLP coatings with coat weights of 12.4 g(CLP)/m^2^ were higher than those of the nonstructured (smooth) commercial coatings used for comparison and similar to that of wood. Still, the root-mean-square height (*S*_q_), which was less affected by the roughness and unevenness of the wood’s structure, increased when the amount of GDE decreased. The GDE likely decreases surface roughness by decreasing the particle’s shape definition and filling voids between them. This was also observed with AFM (Figure 2 and Table 1).

The effect of the coat weight on the surface roughness was examined for samples with a GDE/CLP ratio of 0.52 g/g. Decreasing the coat weight of these samples produced significantly higher WCAs, and thus, it was expected that similar trends would be observed in surface roughness. However, the surface roughnesses of coatings with weights of 12.4, 8.9, and 6.9 g(CLP)/m^2^ were not significantly different from one another. It could be that light interferometry analysis area (236 μm × 315 μm) was too large to properly differentiate the nanoscale roughness caused by the particles from the microscale roughness of wood. No particles were distinguished from the obtained images, supporting this assumption. Similar behavior has been observed by Forsman et al.^36^ We examined an even thicker layer to get some structural insights into the effect of the coat weight on the coated surface. We observed that coat weights of 60 g(CLP)/m^2^ on wooden samples resulted in WCAs of only 93° with a GDE/CLP ratio of 0.52 g/g. While this is a high WCA for a lignin coating, it is significantly lower than that obtained here for thinner layers. Such thick layers leave no empty spaces between the particles and are therefore likely to diminish the roughness-mediated hydrophobic effects of the wood, which makes the surface chemistry dominant. In contrast, a coat weight of 12.4 g(CLP)/m^2^ forms a thin enough layer of particles to maintain the intrinsic macroscopic unevenness of the wood surface (Figures 3c and S6). The results underline the importance of maintaining the surface roughness to achieve hydrophobicity.

These findings are in agreement with earlier studies on nanostructured coatings^29−31,59,60^ and theory.^61^ For example, Forsman et al.^30^ observed that very thin wax particle coatings on textiles provided a significant hydrophobizing effect, which was correlated to the samples’ surface roughness. It was observed that thermal annealing, which melted the particles, lowered the surface roughness and the obtained WCAs. Söz et al.,^31^ who used silica particles, observed that while hydrophobic effects are created by nanoparticle-induced surface roughness, multiple layers of particles can decrease the surface roughness and hydrophobicity when voids between particles are being filled and large aggregates form.

These findings suggest that the particulate element of the coating contributes significantly toward retaining and even increasing the surface roughness of the substrate. When thin layers are used, the surface roughness is maintained giving the coated surface significant water protection. Surface roughness images of the uncoated wood, the other commercial samples, and the GDE/CLP ratios of 0.65 and 0.39 g/g can be found in the Supporting Information (Figure S6).

### 3.4 Moisture Buffering Effect

**Figure 4:**
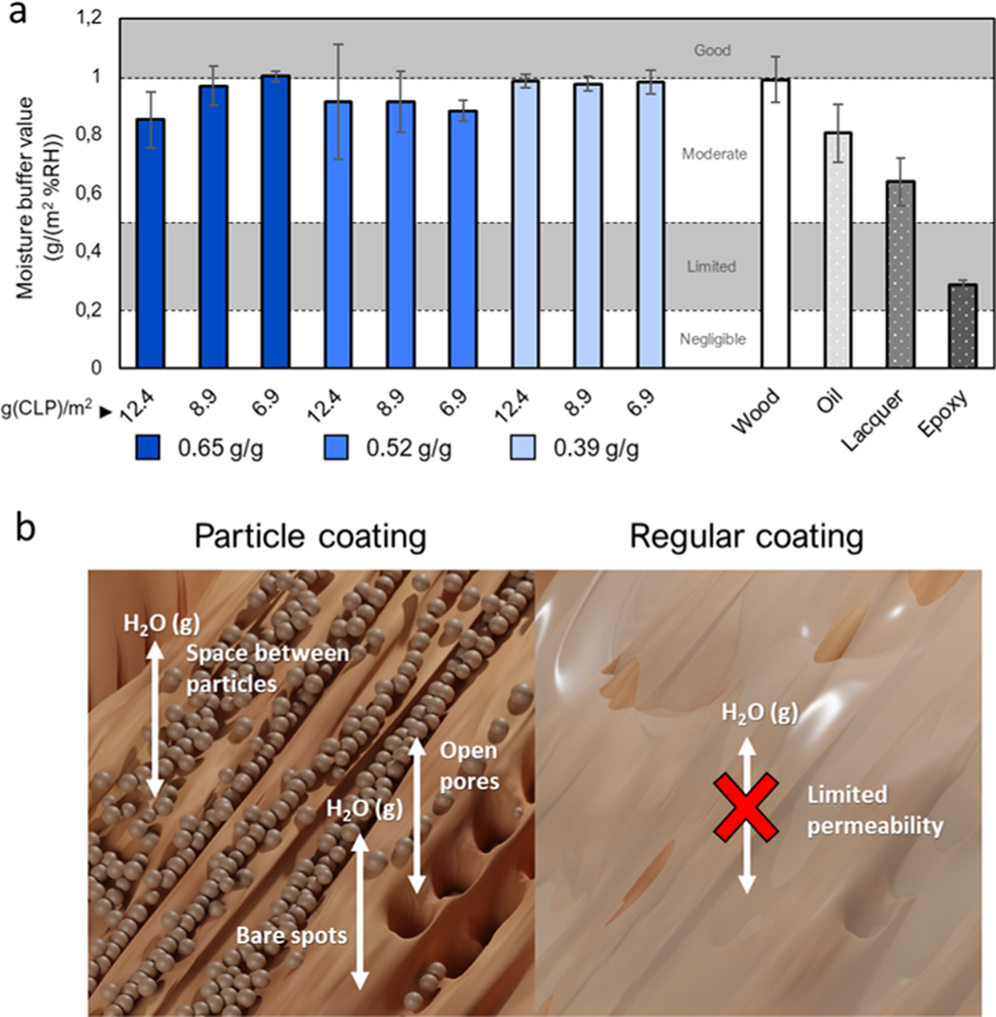
Moisture buffering properties of CLP–GDE coatings. (a) Moisture buffer properties of wood coated with CLP–GDE coatings of three ratios (0.65, 0.52, and 0.39 g GDE/g CLP) and three coat weights (6.9, 8.9, and 12.4 g CLP/m^2^) and commercial reference coatings. (b) Illustrations of the moisture buffering mechanisms of particulate and film-forming coatings. The classifications in (a) are defined according to the NORDTEST method.^52^

The use of hygroscopic (moisture buffering) materials like wood in buildings is beneficial for indoor air quality and comfort because it can moderate the humidity variations occurring due to, *e.g.*, the number of people, heating, or use of water in the indoor spaces.^52,62^ However, film-forming paints or lacquers may diminish this effect. Hence, it is beneficial to develop coatings that can protect the wood while preserving the substrate’s moisture buffering ability. Thus, the moisture buffer values of wood coated with different thicknesses of the GDE/CLP ratios 0.65, 0.52, and 0.39 g/g were evaluated (Figure 4).

Uncoated wood had a moisture buffer value of 1.0 g/(m^2^%RH), which is on the limit to be classified as good.^52^ The GDE/CLP coatings had a slightly negative effect on the moisture buffering ability but decreased the moisture buffer value (MBV) by a maximum of 15%. All GDE/CLP coatings outperformed the commercial references, which all impeded the breathability of the substrate significantly. The oil and lacquer impeded the breathability to 0.8 and 0.6 g/(m^2^%RH), respectively, while the epoxy coating impeded the breathability to 0.3 g/(m^2^%RH). The most probable reason for the very small decrease in MBV for the particulate coatings was the very low coat weight needed to obtain good protective layers. Studies on wax coatings have shown that the thickness of coatings strongly affects the moisture buffering of wood coated with wax films, while particulate wax coatings effectively retain the moisture buffering ability of wood.^38^ A coat weight of 12.4 g(CLP)/m^2^ is less than half the mass of the commercial coatings. Achieving effective protection with such thin layers would be almost impossible with film-forming coatings, showing the advantage of particulate coatings for water-repellent and protective coatings. Thicker layers of 20 g(CLP)/m^2^ and 40 g(CLP)/m^2^ resulted in MBVs of 0.7 and 0.4 g/(m^2^%RH), respectively. Thick, multilayered coatings may hence be beneficial for applications that require moisture barrier properties, such as packaging.

### 3.5 Solvent and Stain Resistance

**Figure 5:**
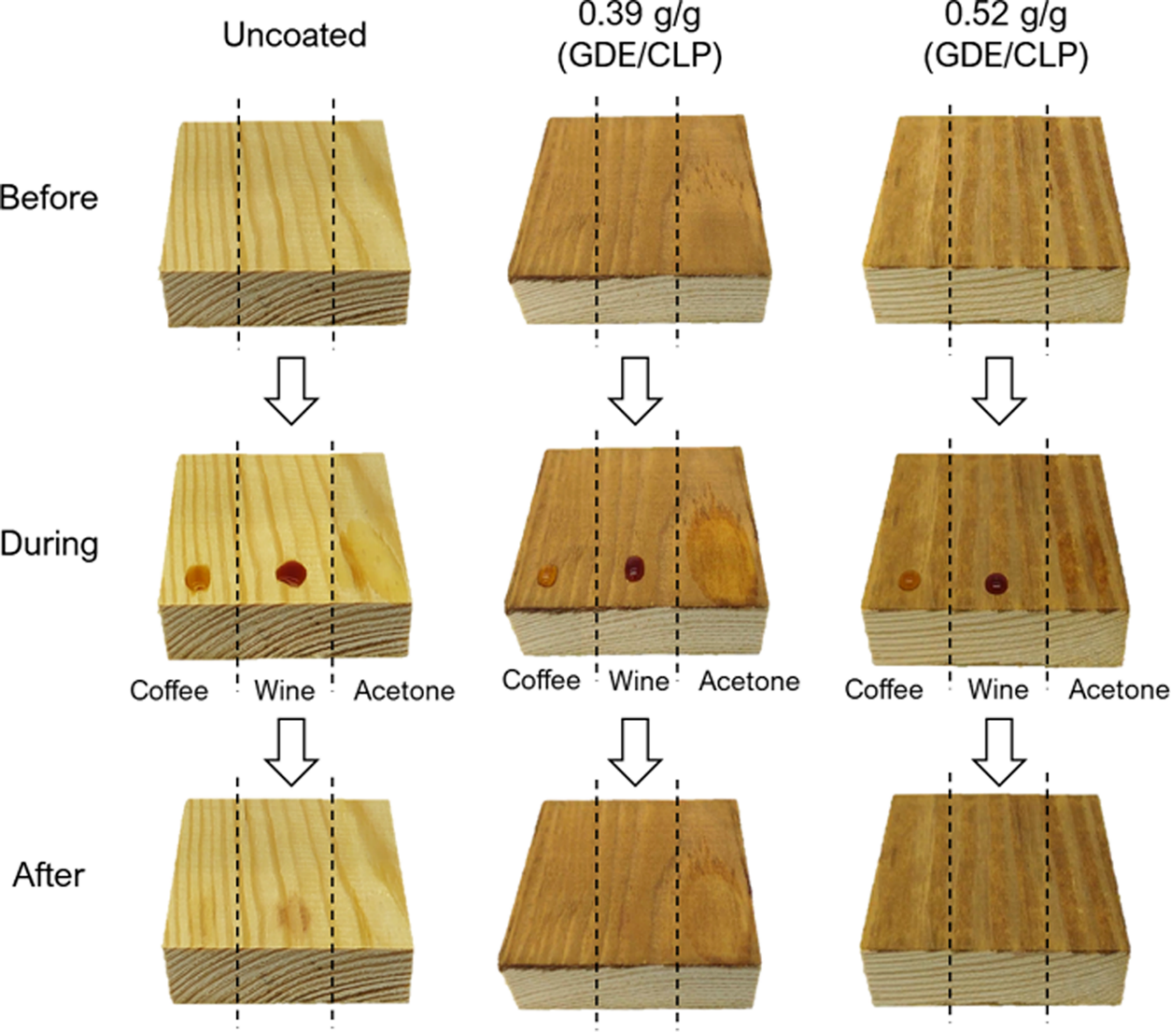
Photographs of uncoated wood and wood coated with GDE/CLP of different ratios with a coat weight of 6.9 g(CLP)/m^2^ before and after being stained with coffee, wine, and acetone, demonstrating their solvent and stain resistance.

Resistance against common staining liquids, such as wine or coffee, is important for domestic applications like wooden furniture, while solvent resistance is often important in, *e.g.*, automotive and aerospace applications. The solvent and stain resistance of the coatings were therefore evaluated using analytical-grade acetone, wine, and coffee. While uncoated wood is easily stained, the stain and solvent resistance of the GDE/CLP ratios of 0.65 and 0.52 g/g and the commercial coatings were excellent. Only the GDE/CLP ratio of 0.39 g/g was damaged by the acetone (Figure 5).

The photographs in Figure 5 show that although the coatings with a GDE/CLP ratio of 0.39 g/g are stain-resistant, the cross-linking is not sufficient to achieve solvent resistance. Acetone produced a permanent stain on that coating. In contrast, the GDE/CLP ratios of 0.65 and 0.52 g/g did not experience any change in appearance due to exposure to acetone. The coatings were, in fact, so resistant to acetone that they could only be removed from metal plates by being forcefully scraped off, as solvents were not able to soften the coatings sufficiently.

### 3.6 Sunlight Resistance

**Figure 6:**
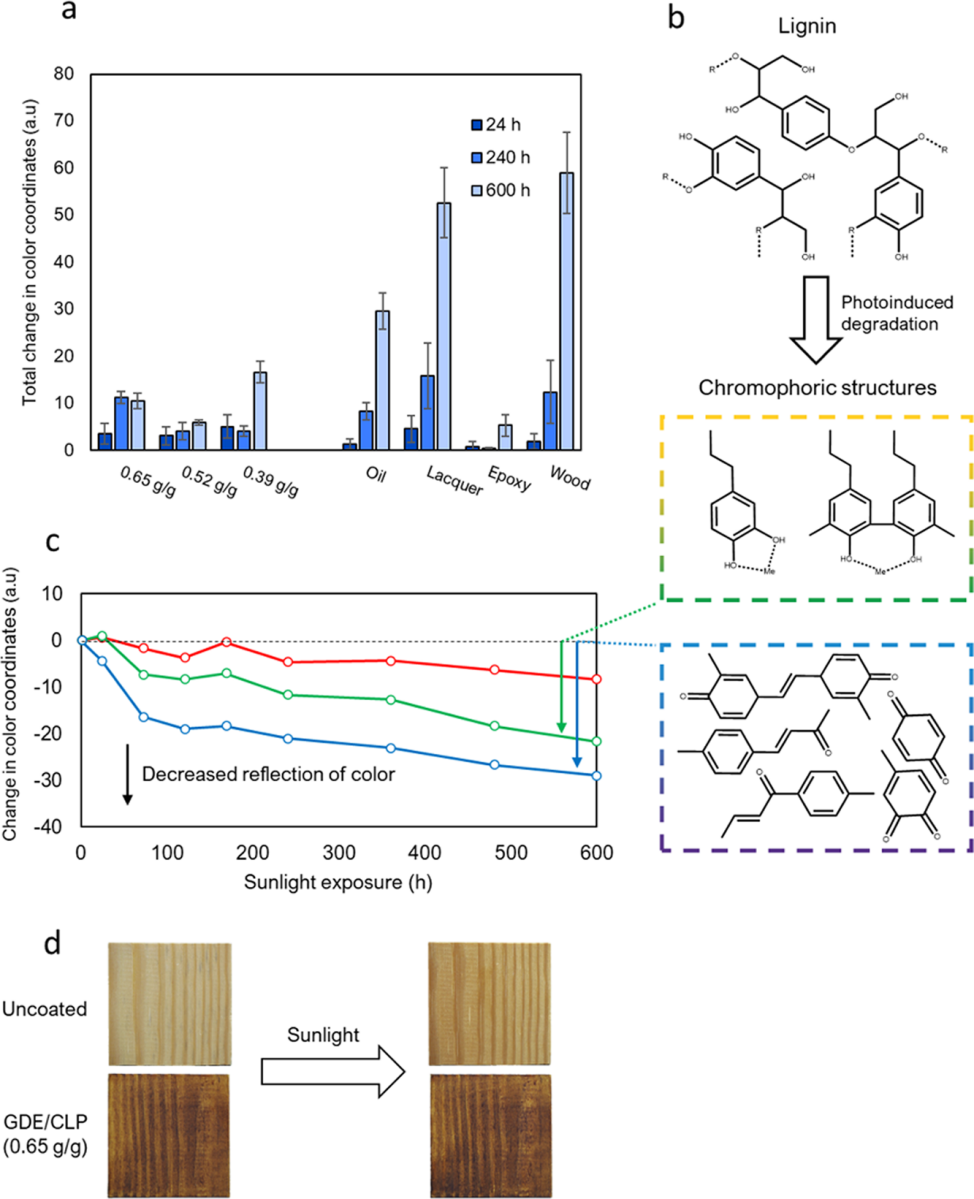
Sunlight-induced color change of coated and uncoated wood due to photoinduced degradation of lignin, simulated using a xenon lamp. (a) Total change in color coordinates of wood coated with GDE/CLP coatings of different ratios with a coat weight of 12.4 g(CLP)/m^2^ and commercial reference coatings, and uncoated wood due to exposure to simulated sunlight. (b) Photoinduced degradation of lignin creating chromophoric structures, (c) which absorb light of green and blue hues.^4,63^ (d) Photograph illustrating the color change of uncoated wood after 600 h of simulated sunlight.

Objects that are regularly exposed to sunlight require resistance against light-induced deterioration in addition to the other previously discussed properties to maintain their appearance and mechanical properties. To evaluate whether the GDE/CLP coatings were prone to sunlight-induced degradation, coatings were exposed to 600 h of simulated sunlight while their appearances were monitored regularly (Figure 6).

Both GDE/CLP coatings with ratios 0.65 and 0.52 g/g were able to reduce the color changes well. In contrast, the sample coated with a GDE/CLP ratio of 0.39 g/g did not retain its original colors as well as the samples coated with the two higher GDE/CLP ratios. The incomplete cross-linking in this ratio, which was also discussed in Sections 3.1 and 3.5, seems to negatively affect the coating’s ability to bond to the substrate since some of the coating had unexpectedly been torn off by the cooling airflow. Wood samples coated with commercial oil and lacquer were also significantly changed by the simulated sunlight. However, the oil coating only changed to a slightly more purple hue, and the color change was hardly noticeable by merely comparing the visual appearance before and after exposure with the naked eye.

The isolated color changes show that light-induced color changes of uncoated wood mostly cause an increase in the absorption of green and blue hues (Figures 6b,c and S7). The changes in light absorption occur due to the generation of chromophoric structures from the photodegradation of lignin,^63^ so the CLP–GDE coatings would intuitively be expected to change in appearance by the same reactions that occur in wood. Structural differences may nevertheless impact the degradation. It has been hypothesized that lignin UV photodegradation begins from phenolic hydroxyl groups.^4^ Since GDE will reduce native phenolic hydroxyl groups (by converting them to ethers) when reacting with them, the lack of these groups could explain the lack of significant color change in the GDE/CLP ratios of 0.65 and 0.52 g/g. The commercial epoxy coating showed excellent resistance against light-induced color change. Nevertheless, the color of the coating was originally white, which naturally decreased the amount of light absorbed by the coating and changed the color of the wood.

### 3.7 Thermal Resistance

**Figure 7:**
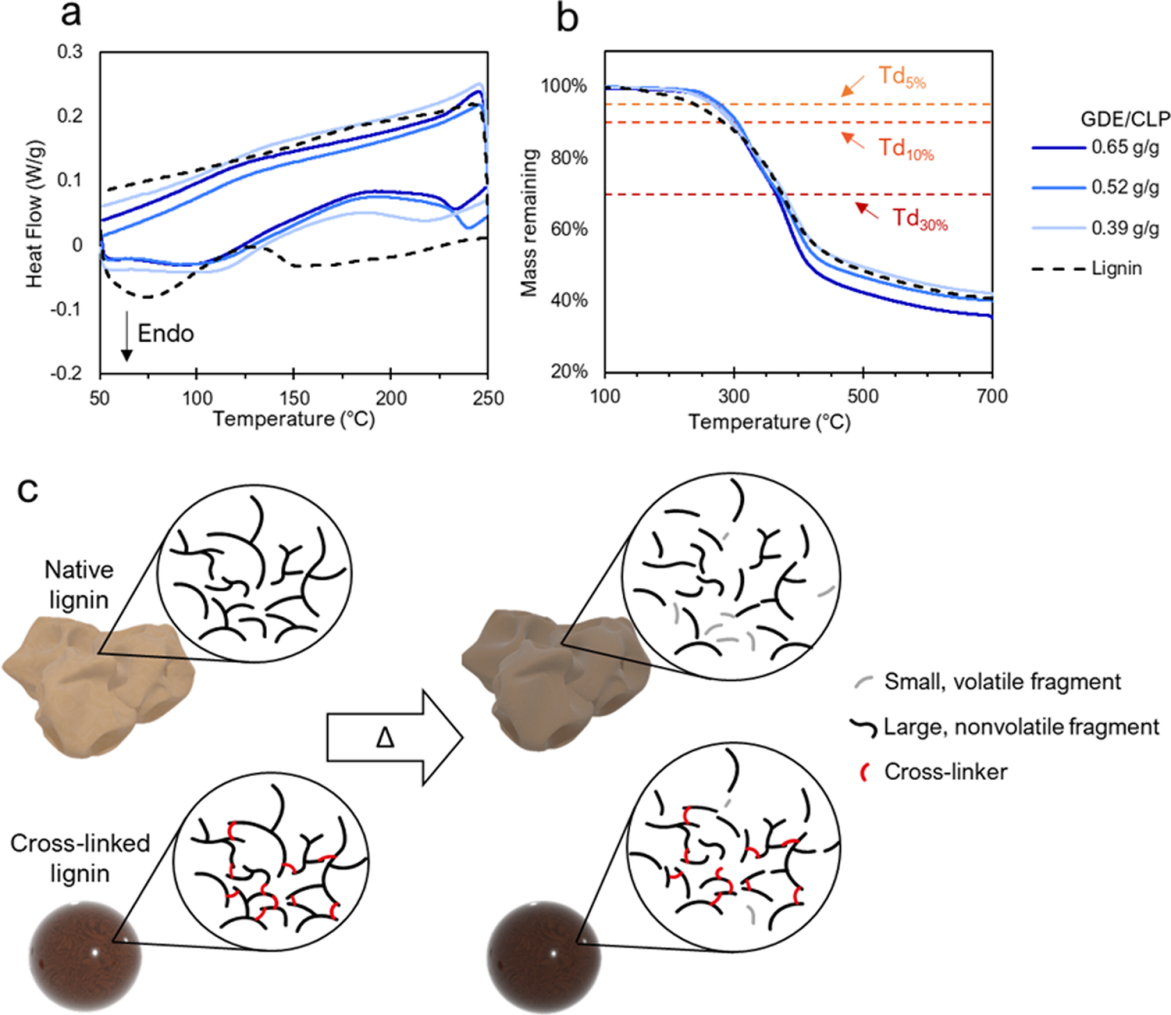
Thermal degradation of unmodified kraft lignin and CLP–GDE networks. (a) DSC and (b) TGA thermograms of lignin and cross-linked coatings. (c) Illustration of the cross-linking mode of thermal stabilization.

Resistance to heat-induced deterioration can in some applications be crucial. Thermogravimetric analysis (TGA) and differential scanning calorimetry (DSC) were used to determine temperatures at which irreversible and reversible temperature-induced changes in the chemical structure of the CLP–GDE coatings take place (Figure 7).

**Figure 8:**
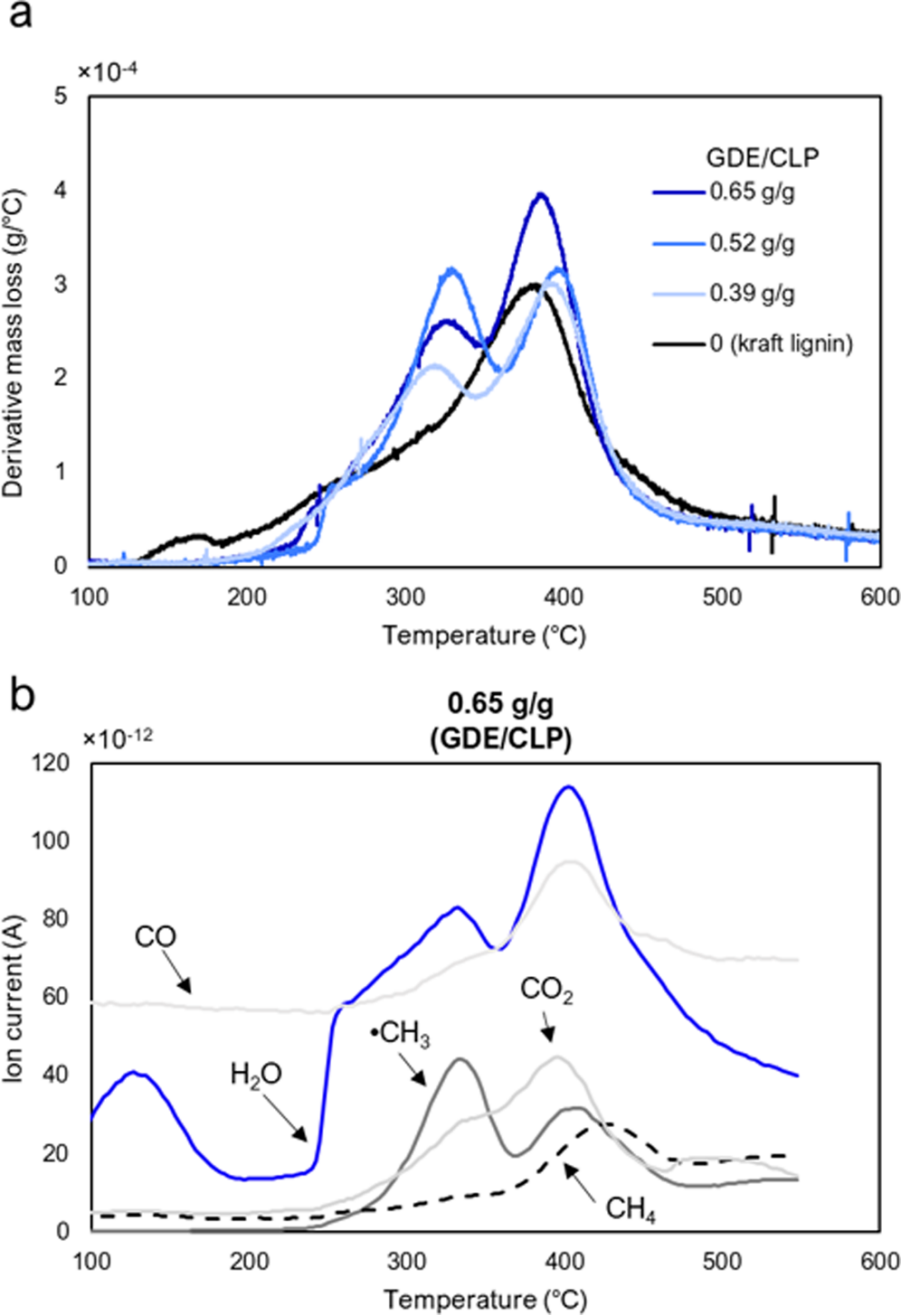
Thermal degradation peaks of lignin and CLP–GDE networks. (a) Derivative thermal degradation of CLP–GDE coatings and lignin measured by TGA and (b) the main gaseous products released by thermal degradation of a GDE/CLP coating with a ratio of 0.65 g/g measured by STA-MS.

Unmodified kraft lignin experienced an endothermic region between 50 and 90 °C and a slight exothermic peak at 135 °C, which is contributed to water evaporating and the glass transition (*T*_g_), respectively.^64,65^ Similar endothermic peaks were observed in the GDE/CLP coatings at 190, 225, and 230 °C for the GDE/CLP ratios 0.39, 0.65, and 0.52 g/g, respectively. The higher *T*_g_ in cross-linked samples is due to the restricted movement of polymer chains within the cross-linked matrix. TGA measurements showed that the GDE-cross-linked lignin samples had two mass loss peaks, the first between 320 and 330 °C (Td_max1_) and the second at 390–395 °C (Td_max2_) (Figure 8a and Table 2). For the GDE/CLP ratios of 0.65 and 0.39 g/g, the latter degradation peak is significantly more intense, while both degradation peaks are similarly intense for the ratio of 0.52 g/g. The latter peak is shared by unmodified kraft lignin, although it occurs at a slightly lower temperature of 385 °C. The initiation of thermal degradation was significantly delayed by the cross-linking, as shown by the higher Td_5%_ and Td_10%_ temperatures indicating where 5 or 10% degradation has occurred. respectively. However, the Td_30%_, indicating the temperature at which 30% of the initial mass has degraded, comes earlier for the samples containing more GDE. The GDE/CLP ratio of 0.39 g/g also had a higher heat resistance index temperature (*T*_HRI_) (Figure 7b and Table 2). These findings indicate that the presence of GDE may negatively affect the heat resistance at high temperatures (over 300 °C) although delaying the initiation of thermal degradation. It is also interesting to note that the GDE/CLP ratio of 0.39 g/g had the highest percentile residue at 700 °C, while the ratio of 0.65 g/g had the lowest (Table 2). Because aliphatic structures are more accessible to thermal degradation compared to aromatic rings,^66^ it is not surprising that the incorporation of a large amount of GDE lowers the percentile residue at high temperatures.

**Table 2:** Differences in Thermal Degradation of CLP–GDE Coatings and Lignin

GDE/CLP (g/g)	Td_5%_ (°C)	Td_10%_ (°C)	Td_30%_ (°C)	*T*_HRI_ (°C)	Td_max1_ (°C)	Td_max2_ (°C)	% residue at 700 °C
0 (lignin)	241	285	347	157		386	40
0.39	269	297	378	164	322	395	42
0.52	279	305	370	163	328	397	40
0.65	269	297	365	160	329	391	35

Simultaneous thermal analysis with mass spectrometry (STA-MS) was used to determine the cause of the peak at 320 °C (Figure 8b). It was observed that the most significant gaseous substances at 320–330 °C were water and methyl radicals, while the latter degradation peak at 390–395 °C consisted of water, carbon monoxide and dioxide, methyl radicals, and methane, in that order. The water released at 320 °C could either be released by the cleavage of hydroxyl groups or from moisture trapped within the material. As the hydroxyl group content, in theory, should remain unchanged after a reaction between a hydroxyl group and an oxirane group, it is possible that moisture trapped within the cross-linked particles is one reason for the increased degradation rate at high temperatures in the GDE/CLP ratios of 0.65 and 0.52 g/g. However, the methyl radical peak at 320 °C also indicates some structural degradation. Methyl radicals may arise from the scission of aromatic methoxyl side chains^67^ or possibly from degradation of the aliphatic glycerol structure, which is incorporated with the GDE.^66^

The combined results of the DSC, TGA, and STA-MS analyses show the positive effect of cross-linking on the thermal stability of the CLP/GDE coatings. Cross-linking generally increases thermal stability by increasing the number of bonds, and consequently energy, required to volatilize fractions of the polymer chains.^66^ Previous studies on lignin-epoxy thermoset composites have reported similar improvements in thermal resistance by cross-linking.^68^ Dense polymer chain packing can also positively affect the thermal stability of materials.^69^ However, in contrast to homogeneous resins such as epoxies or polyurethanes, lignin is a bulky and heterogeneous noncrystalline polymer, which makes it difficult to determine the effect of chain density or voids introduced by the cross-linker. Lignin’s polyaromatic structure is nevertheless chemically rather stable,^67^ which is reflected in its high percentile residual mass at 700 °C. To compare with another particulate coating, we note that coatings containing silica nanoparticles in an epoxy matrix of BPA and epichlorohydrin reached Td_5%_ and Td_max_ of 150 and 355 °C, respectively, and a residual mass of 11.6% at 700 °C.^70^ The thermal resistance found for the CLP-based coatings is suitable for most indoor and outdoor applications, although extreme conditions requiring long and/or regular exposures above 250 °C may initiate significant degradation and should therefore be avoided.

## 4 Conclusions

In this study, the amphiphilic properties of CLPs were exploited in a novel manner to prepare water-based, solvent-free, and multiresistant surface coatings for rigid surfaces such as wood and metal. The effects of the GDE/CLP ratio, coat weight, and curing time were evaluated to understand the network formation mechanism and to find the best system for optimum performance. Due to their hydroxyl groups, the CLPs acted as hardeners and required no binder to adhere to the substrate. The coatings with GDE/CLP ratios of 0.52 and 0.65 g/g withstood solvents, stains, abrasion, heat, and light while also being breathable and water-repellent. The particle morphology allowed for efficient water repellency with low coat weight since the coating preserved the surface roughness of the wooden substrate while providing additional hydrophobicity. Furthermore, the natural moisture buffering ability of wood was significantly less impeded by the CLP–GDE coatings compared to that of commercially available coatings (oil, lacquer, and epoxy), which is attributed to both the particle morphology and the low coat weight.

This work demonstrates, for the first time, the preparation of fully particulate coatings without the use of a binding matrix using lignin instead of metal oxides. This work not only presents a durable and scalable particulate coating^23,24,26^ but also broadens the field of particulate coatings to the domain of lignin particle technology.

## Supporting Information Available

The Supporting Information contains two-dimensional AFM images and additional SEM image of coating; dynamic light scattering size distributions of CLP dispersion, full FTIR spectra of coatings with a GDE/CLP ratio of 0.52 g/g and narrow spectra from curing monitoring test; tensiometer images showing water adsorption through coatings; interferometry surface roughness profiles; individual color change by simulated sunlight; and P^31^-NMR results (PDF)
