# Supplementary material for: Colloidal Lignin Particles and Epoxies for Bio-Based, Durable, and Multiresistant Nanostructured Coatings
Source: ACS Appl Mater Interfaces. 2021 Jul 15;13(29):34793–806. doi: 10.1021/acsami.1c06087 (PMC8397241; doi:10.1021/acsami.1c06087)
Supplement: Supplementary file 1 — am1c06087_si_001.pdf [file am1c06087_si_001.pdf]

Supporting Information

Colloidal Lignin Particles and Epoxies for Bio-based, Durable, and Multiresistant Nanostructured Coatings

Karl Alexander Henn[a], Nina Forsman[a], Zou Tao[a], Monika Österberg[a]*

[a] Aalto University, School of Chemical Engineering, Department of Bioproducts and Biosystems, Vuorimiehentie 1, 02150 Espoo, Finland (firstname.surname@aalto.fi)

* Correspondance to Monika Österberg (monika.osterberg@aalto.fi, Vuorimihentie 1, 02150, Espoo, Finland)

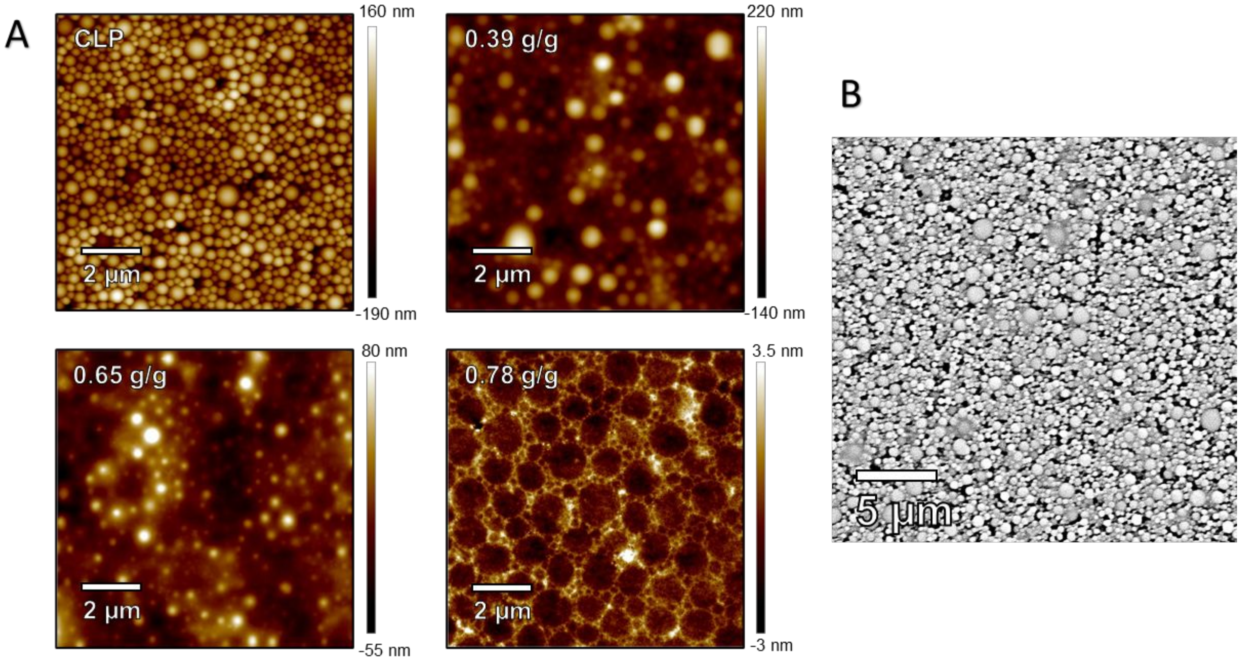

Figure S1. Microscopy images of coatings with different GDE/CLP ratios. (A) AFM images of CLPs and GDE/CLP mixtures of different ratios (0.39, 0.65, 0.78 g/g). (B) An SEM image of GDE/CLP mixture with the ratio of 0.65 g/g. All samples were cured for one hour at 105°C.

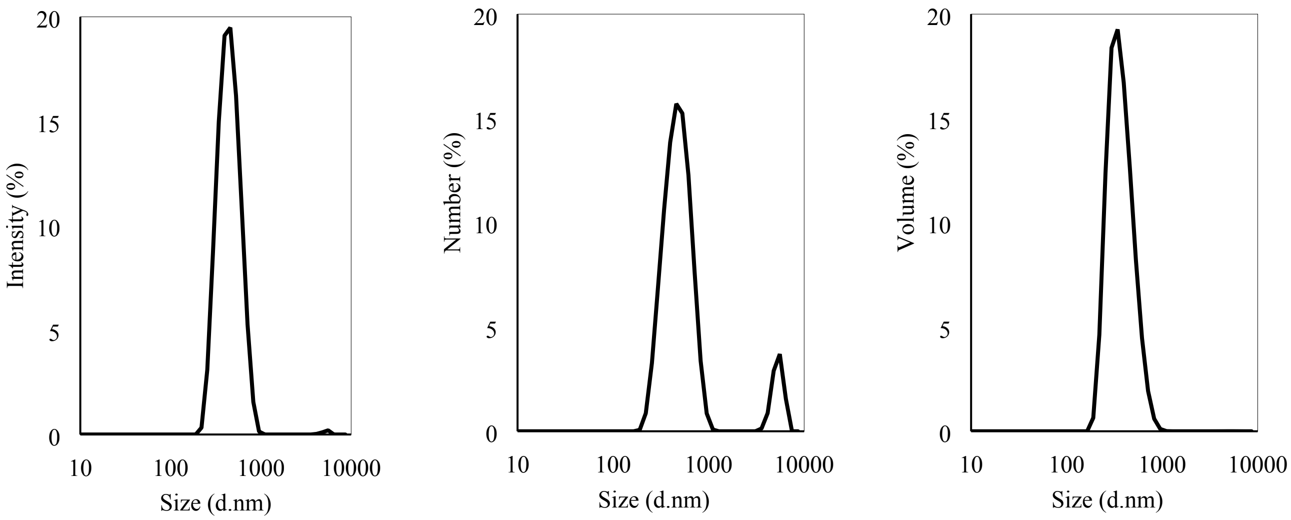

Figure S2. Dynamic light scattering particle size distributions of CLPs.

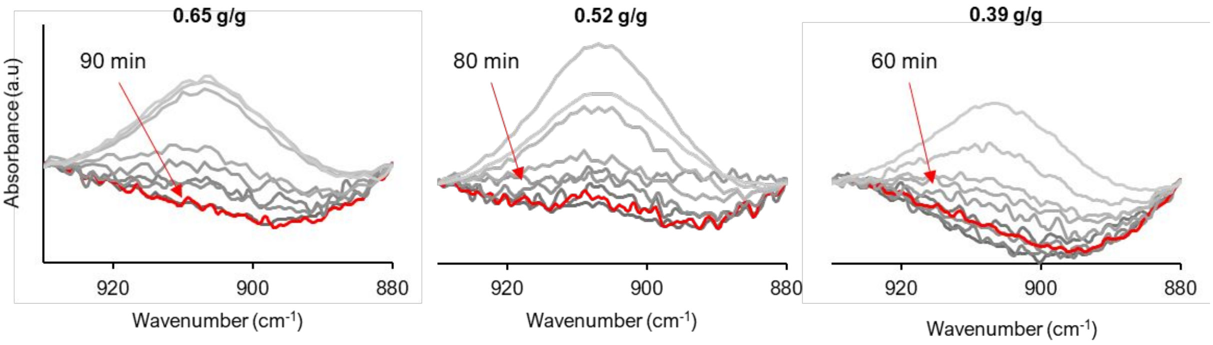

Figure S3. Normalized FTIR absorbance intensities between wavenumbers 880 – 930 cm^{-1} of coatings with different GDE/CLP ratios at different curing times (10 – 100 minutes, from light to dark hue). The red line signifies the first signal whose intensity is below 8% of its original intensity, signifying a near completed curing.

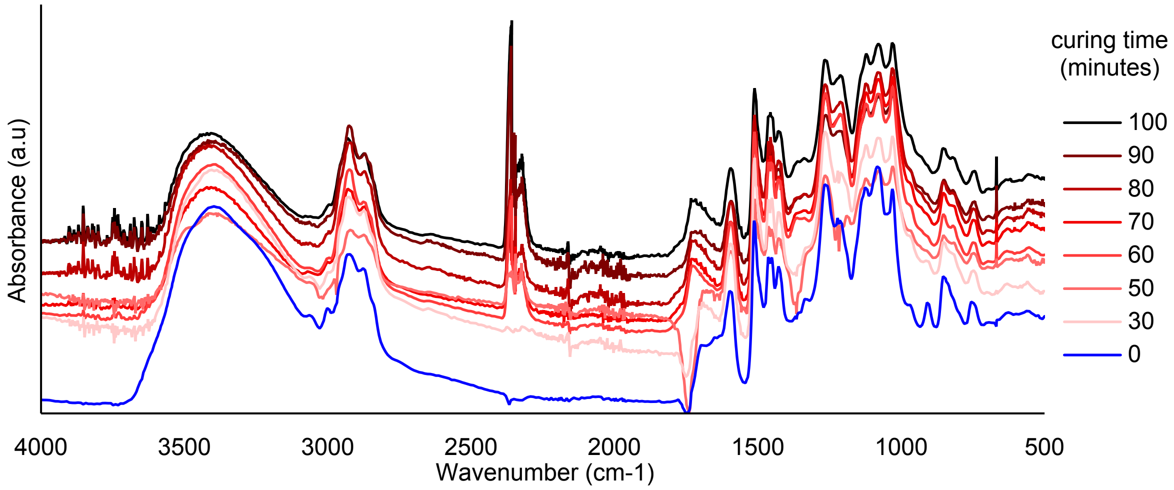

Figure S4.FTIR spectra of coating with the GDE/CLP 0.52 g/g cured for different durations at 105°C.

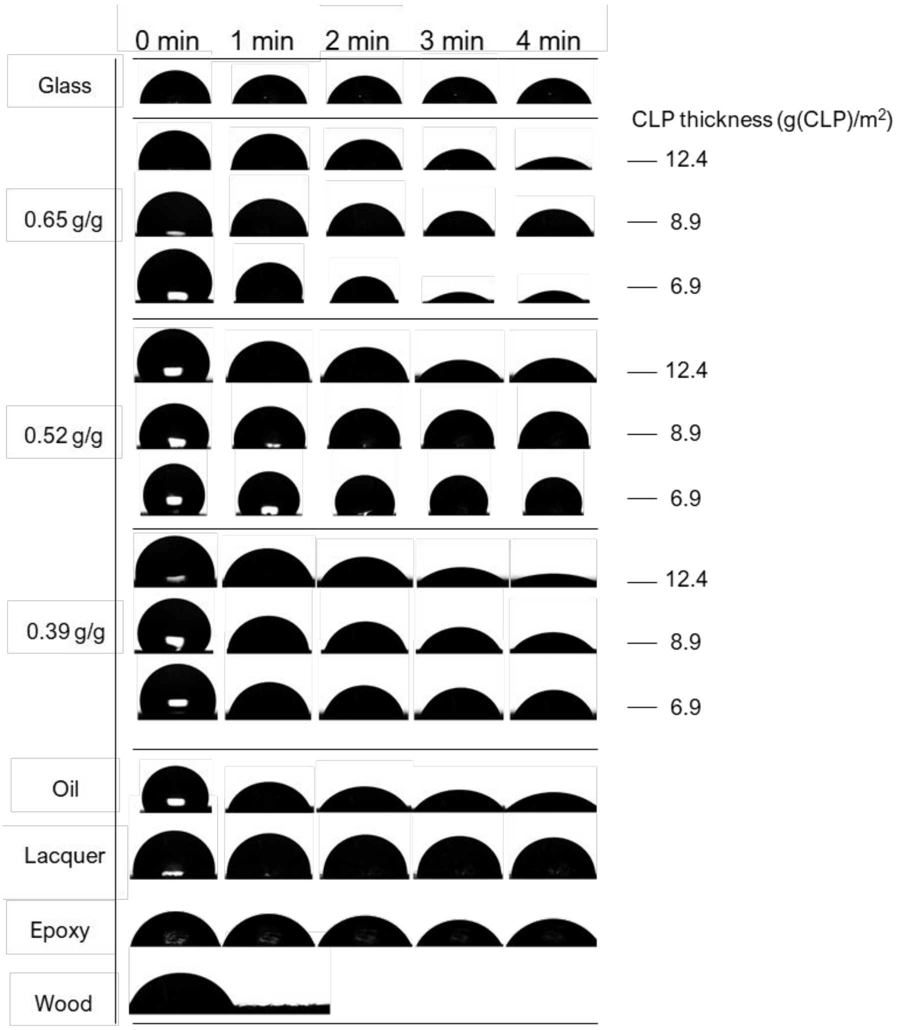

Figure S5. Change in volume of water due to absorption through GDE/CLP coatings of different thicknesses (12.4, 8.9, 6.9 $g(CLP)/m^2$, thickest to thinnest) and through wood oil, commercial epoxy, wood lacquer, and uncoated wood.

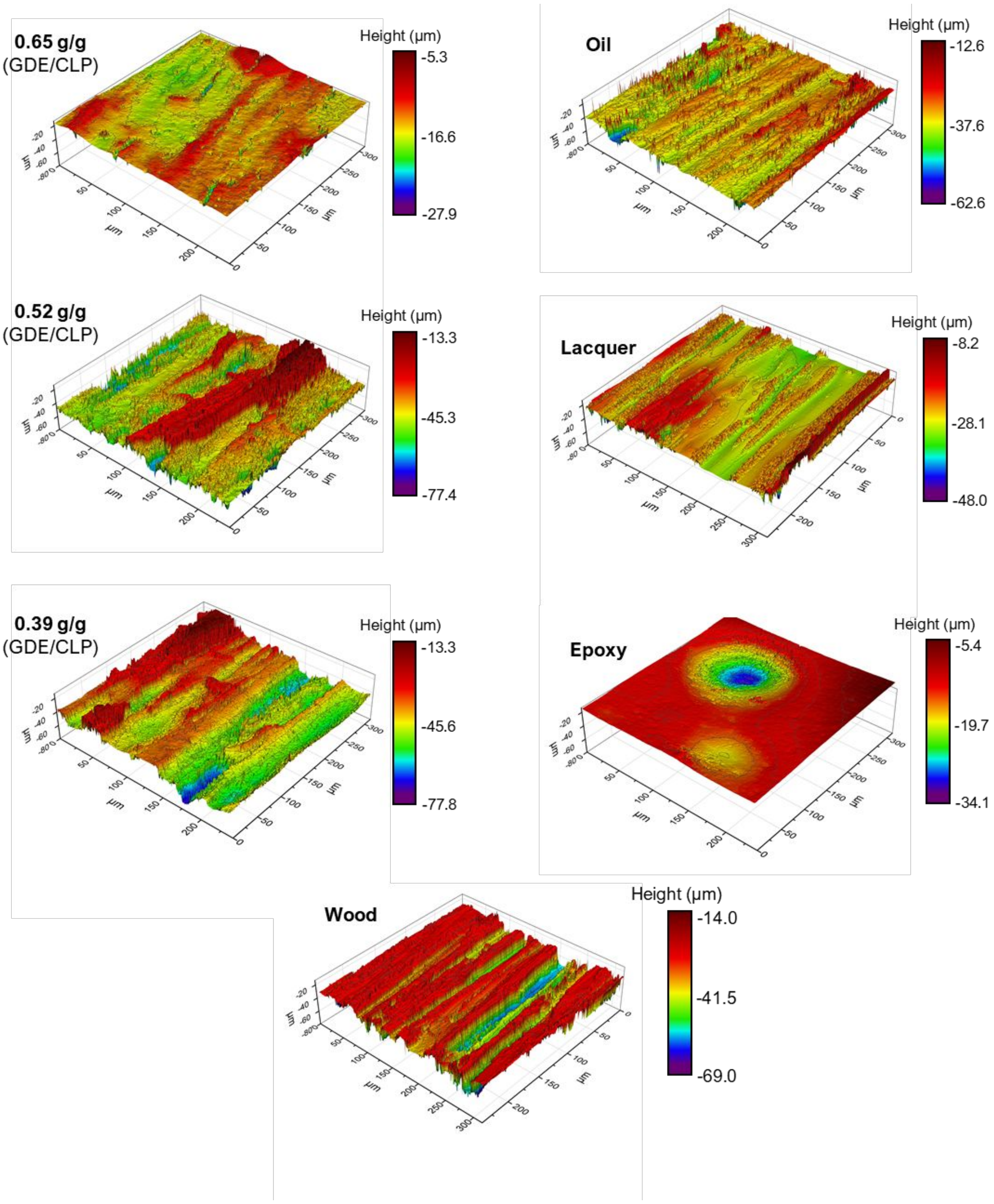

Figure S6. Light-interferometry images of wood coated with GDE/CLP coatings with thicknesses 12.4 g(CLP)/m^2 and different GDE/CLP ratios and reference coatings.

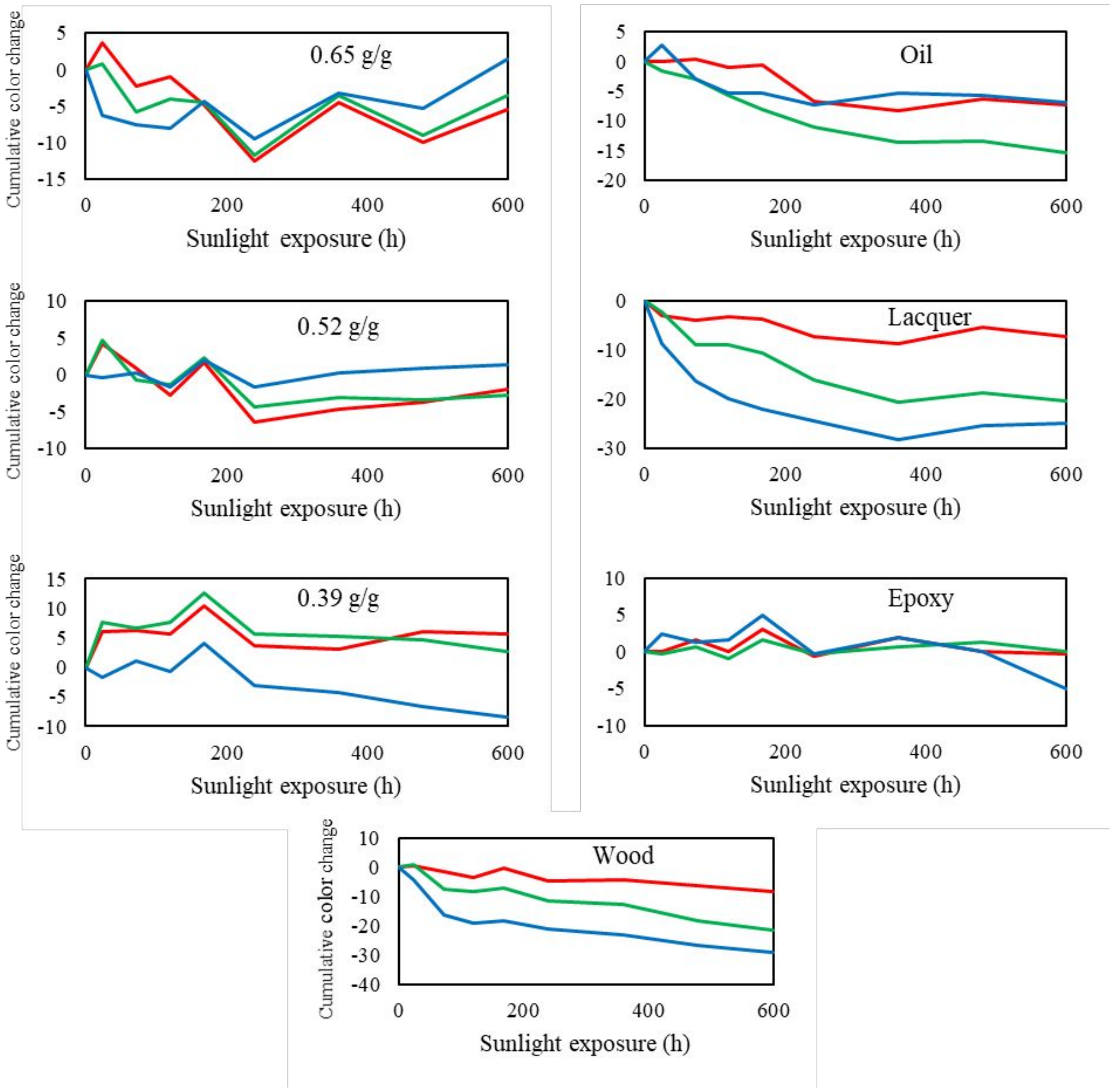

Figure S7. Isolated color change of GDE/CLP coatings with the thickness 12.4 g(CLP)/m^2, reference coatings, and uncoated wood by exposure to simulated sunlight.

Table S1. ^{31}P-NMR characterization results of UPM Biopiva 100 kraft lignin for the determination of hydroxyl content.

Sample no.	Aliphatic OH (mmol/g)	Phenolic OH (mmol/g)	Carboxylic OH (mmol/g)	Total OH (mmol/g)
1	1.95	3.85	0.45	6.25
2	2.17	3.87	0.48	6.52
